# Letter from the Program Directors

**DOI:** 10.19102/icrm.2024.15038

**Published:** 2024-03-15

**Authors:** Wendy Tzou, William Sauer


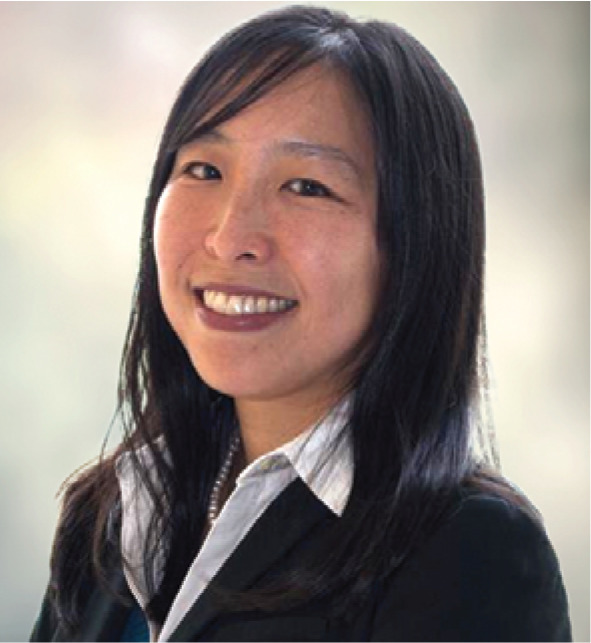



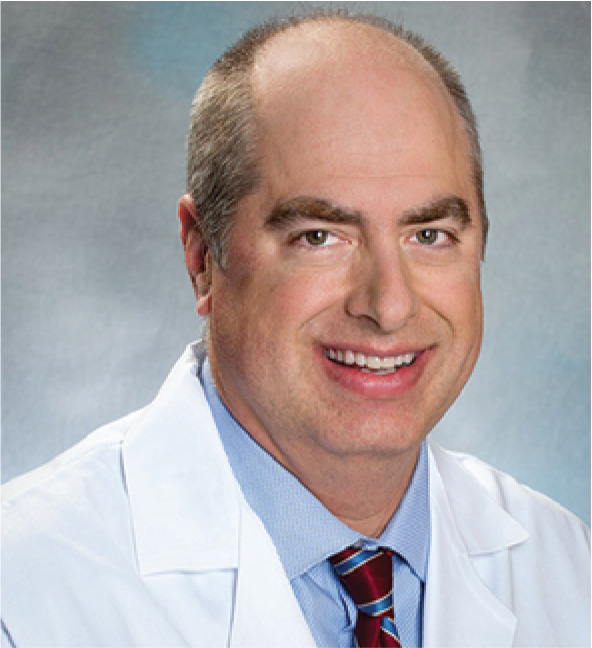


Dear readers,

As the Directors for the Electrophysiology Fellows Summit and the Cardiac Arrhythmia Scholars Program, we are proud to introduce the published case reports of the three finalists who were selected to present their unique cases during the Fellows Competition session at the Electrophysiology Fellows Summit in November 2023.

After a thorough review of the numerous exceptional case entries submitted by fellows and residents from around the globe, the program committee nominated these finalists to present their work and participate in panel discussions, with the overall winner announced at the sessions’ conclusion following the panel deliberation. (For those who were unable to attend or who wish to view the sessions again, EP Fellows Summit ON-DEMAND at www.epfellowssummit.com provides instant and unlimited access to the full library of educational programming from the Summit.)

Dr. Deepti Ranganathan, a fellow at the Sunnybrook Health Science Centre in Toronto, ON, Canada, described a case of recurrent syncope and cardiac arrest, ultimately found to be due to repetitive moderator-band premature ventricular complexes triggering idiopathic ventricular fibrillation. Following mapping and ablation of the moderator-band premature ventricular complexes, there was complete elimination of subsequent ventricular arrhythmias, as confirmed by a subcutaneous implantable cardioverter-defibrillator. This case highlighted the challenging management of a patient with ventricular fibrillation triggered by uniform morphology premature ventricular complexes.

Dr. Connor Oates, a fellow at Georgetown University Hospital Center in Washington, DC, described a rare case of a pregnant patient presenting with multifocal ectopic Purkinje-related premature contractions and cardiomyopathy, who was ultimately found to have a *SCN5A* mutation and in whom treatment with quinidine was an effective strategy for decreasing ectopy, with an acceptable risk profile for use in pregnancy. This case highlights multiple complexities in medical decision-making, especially with respect to the management of arrhythmias in pregnancy.

Dr. Robert Kerley, a fellow at Mater Misericordiae University Hospital, in Dublin, Ireland, presented a case on inappropriate sinus tachycardia refractory to medical therapy and invasive treatments, the latter of which included catheter-based sinus node modification with radiofrequency ablation (limited by phrenic nerve proximity and potential injury) and surgical right cardiac sympathectomy. Pulsed-field ablation in the region of interest in the high right atrium and superior vena cava, over two procedures, ultimately ensured control of inappropriate sinus tachycardia without phrenic nerve injury. This case highlights the potential utility and safety of the use of pulsed-field ablation for effective arrhythmia treatment and a reduced risk of collateral damage.

Congratulations to Dr. Ranganathan on her winning case and to Drs. Oates and Kerley as the case competition finalists for their unique and interesting case presentations.

We look forward to your attendance at the 2024 EP Fellows Summit scheduled for November 1–3, 2024. As a hybrid conference, attendees will have the choice of attending the Summit virtually or as a traditional in-person event in Washington, DC, with the chance to participate in hands-on training sessions and interpersonal engagement. For those who are unable to attend the Summit, virtual attendance and engagement will be made possible from the convenience of your computer or mobile device through the Summit’s innovative livestream broadcast platforms. Detailed information will be available at www.epfellowssummit.com.

Sincerely,

Wendy Tzou, md

University of Colorado Anschutz Medical Campus

Aurora, CO, USA

and

William Sauer, md

Brigham and Women’s Hospital

Boston, MA, USA

